# Quality indicators in undergraduate palliative medicine education: a scoping review

**DOI:** 10.1186/s12909-026-09232-5

**Published:** 2026-04-24

**Authors:** Nia Edwards, John E. Ellershaw, Stephen Mason

**Affiliations:** 1 School of Medicine - Cedar House, University of Liverpool, Ashton Street, Liverpool, L69 3GE England; 2 Palliative Care Unit, Department of Cardiovascular and Metabolic Medicine, University of Liverpool, Room G20a William Henry Duncan Building, 6 West Derby Street, Liverpool, L7 8TX England

**Keywords:** Education, End of Life, Junior Doctors, Quality Indicators, United Kingdom

## Abstract

**Background:**

Palliative care education in undergraduate medical curricula has historically lacked structured outcomes and measurable indicators, resulting in significant variability in the knowledge, skills and confidence that junior doctors possess. This scoping review, conducted in accordance with the JBI Guidelines, aimed to map the evidence on potential quality indicators (QIs) for palliative medicine education in the UK.

**Methods:**

A scoping review was conducted using the JBI methodology. Online databases (Medline, Embase, Emcare and CENTRAL) and grey literature sources were searched for relevant papers. The inclusion criteria were studies that involved undergraduate medical students or foundation-year doctors, focused on quality assessment in palliative care education, set in the UK, and published in English between February 2022 and March 2026. Data extraction and narrative synthesis were used to develop key themes, from which measurable QI statements were formulated. The PRISMA-Scr guidelines were followed in reporting this review.

**Results:**

Ten publications were included. Analysis identified six thematic areas: (1) compulsory palliative care education in curricula, (2) mandatory assessment, (3) simulation-based education, (4) inter- and multi-disciplinary team (MDT) working, (5) clinical placements in palliative care settings, and (6) the use of e-learning and IT. From these themes, six measurable QIs were developed and structured using the Donabedian framework.

**Conclusions:**

The collated evidence highlights the need for a standardised, robust approach to palliative medicine teaching. The proposed QIs provide a practical, measurable framework for curriculum development and quality assurance. Future work should focus on piloting these indicators and evaluating their impact on the preparation of undergraduate doctors for practice.

## Background

Integrating end-of-life practices into the health care system is necessary to maintain human dignity and facilitate the fundamental human right to health at all stages of life [1]. In England and Wales, by 2040, it is expected that at least 160,000 more people will have palliative care needs each year [2]. Foundation-year doctors often provide such care, on average, for 40 patients who die and an additional 120 patients in the final months of life [3]. However, research overwhelmingly suggests that junior doctors feel “unprepared and out of their depth” when caring for those approaching the end of life, especially in ‘out-of-hours’ situations [4]. Billings and Block report that the current training and education in medical school on palliative care “is inadequate, most strikingly in the clinical years” [5]. In the UK, training in palliative medicine is not standardised across medical schools, with research studies indicating that junior doctors frequently require additional training in end-of-life care [4]. In 2014, the World Health Assembly passed its first global resolution on palliative care, WHA67.19, urging the WHO and Member States to enhance access to palliative care and palliative care education, as an essential part of the health system [6]. Since then, only two actionable indicators specific to education have been proposed, as presented in the WHO Handbook for Guideline Development 2022 [7]. A recent systematic review conducted by J Kaypee aimed to identify a more detailed set of quality indicators (QIs) to be utilised in developing undergraduate curricula [8]. Kaypee’s study, running from 01/01/1999 to 14/02/2022, proposed six QI statements and focused on English-language papers from the UK, Canada, New Zealand, and Australia [8]. This study seeks to update and build on the original review by examining the evidence base from 01/02/2022 to 25/03/2026. Given the broad background of potential QIs for undergraduate palliative education, a scoping review was selected as an appropriate method, providing a mapped overview of themes and clarifying existing concepts.

### Review question

The ‘PCC’ framework for scoping review questions, as outlined by the Joanna Briggs Institute [9], was used to develop the research question: What quality indicators, explicit or implicit, exist in the literature on undergraduate and foundation medical education in palliative care in the UK?

- Population: undergraduate medical students and foundation year doctors.
- Concept: actionable quality indicators that could be integrated into curricula.
- Context: medical schools in the United Kingdom.

In developing Quality Indicators, this paper applies the Donabedian model of quality of care [10], an established framework for evaluating health services that underpins recommendations for healthcare improvement. Donabedian’s theory [10] suggests that structure measures (physical and organisational characteristics) influence process measures (care delivered to patients), which, in turn, influence outcome measures (effects on patients and populations) [11]. The findings from this review have been synthesised and refined to inform a practical approach to applying QIs in both clinical and non-clinical contexts.

## Methods

### Initial search and concept table

An initial, limited search of Medline was conducted to identify relevant articles and to determine keywords for developing a comprehensive search strategy. A concept table (Table 1) was used to structure the question and generate a list of keywords related to the scoping review.

**Table 1:** Concept table for scoping review of quality indicators in palliative medicine education in the UK

Concept 1: palliative care	Concept 2: education	Concept 3: doctors
Palliat*	Education	'Foundation year doctor’
‘Palliative care’	Learning	‘Foundation year’
‘Care of the dying’	Teaching	FY1
‘End of life’	Undergraduate	FY2
‘Terminal’	Postgraduate	'Newly qualified doctor'
Hospice	Curriculum	‘New doctor’
	Indicator	‘Doctor in training’
	Training	Fellowship
	Academics	‘Clinical fellow’
	Assessment	‘Clinical fellowship’
	Schooling	Resident
	Study	Registrar
	Studying	‘Medical student’
		‘Student doctor’
		Intern
		‘House officer’
		‘Studying Medicine’

### Search strategy

Search strings were developed from the concept table and applied to the databases Medline, Embase, Emcare, and the Cochrane Central Register of Controlled Trials (CENTRAL), in accordance with the recommendations of the Cochrane Library Handbook [12]. Inclusion criteria were: participants - medical students and foundation year doctors (FY1 and FY2) within education and/or training in palliative care; concept - research papers focused on quality assessment in relation to palliative care education and commensurate with the Donabedian Model [10]; context - studies based in the UK, studies published in English and within the timeframe 01/02/2022 and 25/03/2026. The inclusion criteria were selected to add, publish, and update the work of Kaypee and colleagues to improve the relevance and timeliness of the research [8]. To ensure that all available texts were sourced, selected subject headings (MeSH terms) with free-text keyword terms were used with the Boolean operators ‘AND’ for between concepts and ‘OR’ for phrases within concepts. The exclusion/inclusion criteria were applied, ensuring that only papers that strictly met these requirements were considered for review, with a view to inclusion in the final sample. The search strategy was adapted for each included database (see supplementary material).

### Study/source of evidence selection

**Fig. 1:**
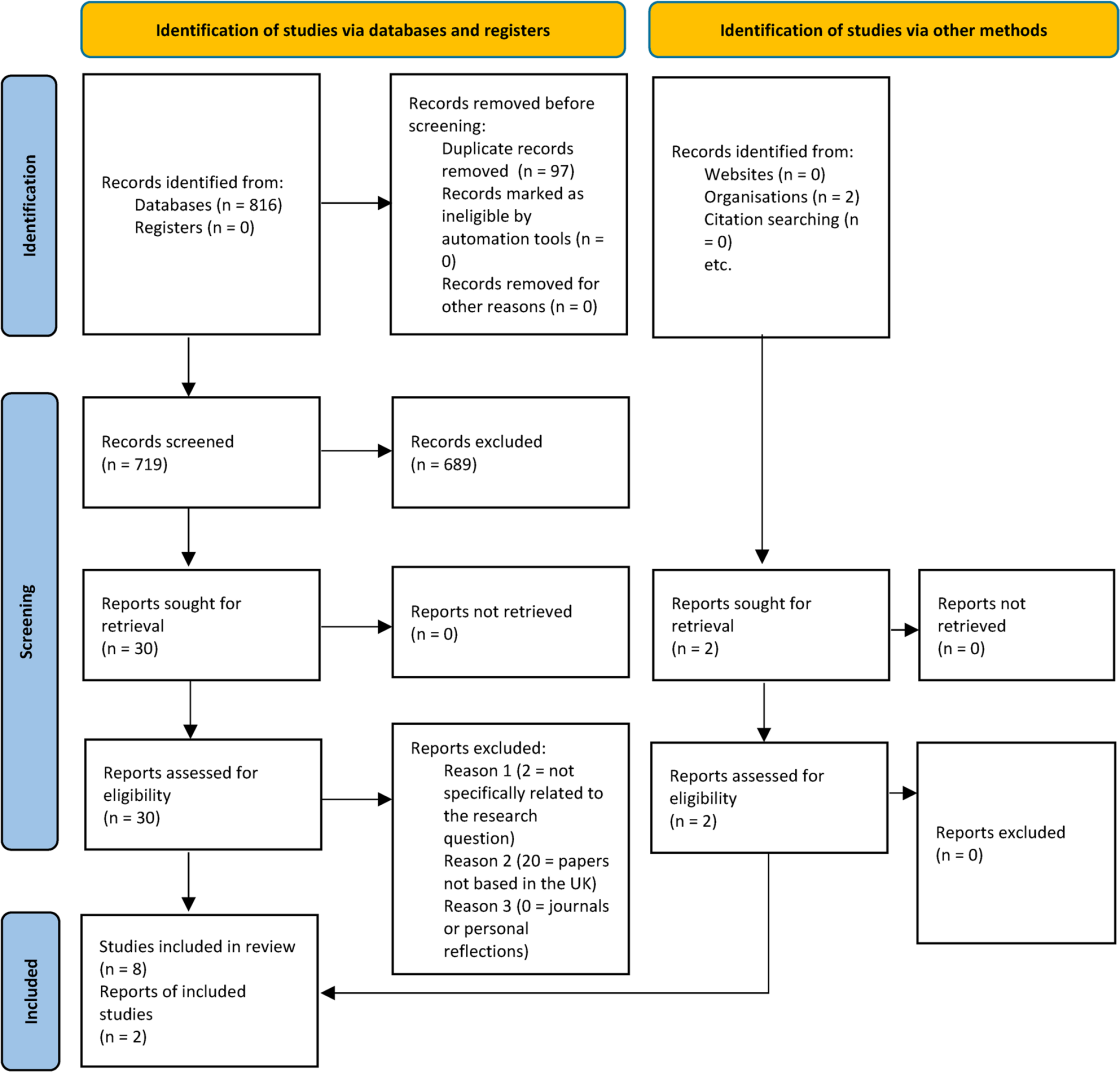
Adapted Prisma diagram for scoping review of quality indicators in palliative medicine education in the UK [13]

Following the search strategy, all identified citations were collated and uploaded to EndNote 21, and duplicates were removed. The results were screened by the research team for eligibility against the inclusion criteria and for relevance to the title of the scoping review, as determined by the lead researcher from the title and abstract. To ensure fidelity and consistency in the application of the inclusion criteria and data extraction process, a subset of the included studies (*n* = 6) was independently reviewed by a senior academic supervisor. Discrepancies or uncertainties were discussed and resolved through consensus. This approach balanced rigour with feasibility, given the scope and timeline of the review. The search and screening results were then incorporated into the final scoping review and are presented in Fig. 1 as an amended PRISMA flow diagram.

### Data extraction and analysis

The data was reviewed and extracted from the papers to include specific details about the participants, concept, context, study methods and key findings relevant to the review question (Table 2) to facilitate comparison and analysis. A narrative synthesis approach was employed, in discussion with J Ellershaw and S Mason, using a charting table to collate all applicable findings and conclusions from the studies, quoting any direct qualitative findings; a summarised version of this is shown in Table 2. This collaborative process facilitated the synthesis, analysis, and summary of findings from the included studies and determined the paper’s relationship to, and relevance for, the research question and PCC context. This method enabled the integration of diverse study designs and identified potential areas for QI development by organising the data into coherent thematic categories. Key concepts and patterns were identified through iterative reading and comparison of study findings, and grouped into overarching themes that reflected the focus of the research question and the nature of the evidence. The synthesis focused on describing how the quality of education and training can be assessed in undergraduate and foundation training in palliative medicine.

**Table 2:** Table summarising the results of the scoping review of quality indicators in palliative medicine education in the UK [15–24]

Title	Authors	Year of publication	Type of study	Countries of origin	Aims/ purpose	Population	Concept	Context	Outcomes related to quality indicators (QIs) in palliative care education
The effect of teaching methods in palliative care education for undergraduate nursing and medical students: a systematic review	Hokka, M. Rajala, M. Kaakinen, P. Lehto, J. T. Pesonen, H. M.	2022	Systematic review	United Kingdom United States Taiwan Iran Korea Spain Germany	To synthesise evidence of the effect of different teaching methods used in palliative care education on students’ competences, knowledge, attitudes, or skills.	Undergraduate nursing and medical students only.	Assessment of any teaching or learning outcome concerning students’ palliative care competencies, knowledge, attitude or skills.	N/A - wide variety of countries studied	Five key potential QI's were developed, which included the effects of: multidimensional interventions, elective courses, simulation, web-based and gaming interventions, interdisciplinary and problem-based education.
Instruments to measure skills and knowledge of physicians and medical students in palliative care: A systematic review of psychometric properties	Lopez-Garcia, M. Rubio, L. Martin-de-las-Heras, S. Suarez, J. Perez-Carceles, M. D. Martin-Martin, J.	2022	Systematic review	United Kingdom Japan Spain United States Austria Italy Kuwait Brazil	To evaluate the psychometric properties of knowledge and skills questionnaires used in palliative care, validated by physicians or medical students based on the Consensus-based Standards for the selection of health Measurement Instruments (COSMIN) methodology.	• Four studies were conducted by general physicians • Four studies in both physicians and nurses • Three studies were in medical students • Two studies were conducted by paediatric specialists	To identify and describe the validated instruments for measuring knowledge and skills around palliative care among physicians and medical students, as well as assess the psychometric properties of these instruments based on the COSMIN methodology.	N/A - wide variety of countries studied	The study found that assessing the knowledge and skills of physicians and medical students in palliative care is essential. It has been shown that self-assessment of acquired competencies can develop physicians’ and nurses’ palliative care skills and improve the quality of patient care. Questionnaires with better psychometric properties, higher scores, and more thorough analysis provide greater reliability and repeatability in this self-assessment.
Online learning in palliative care education of undergraduate medical students: a realist synthesis	Martucci, G. Pereira, J. Busa, C. Centeno, C. Csikos, A. Franchini, L. Elsner, F. Raccichini, M. Mihailescu-Marin, M. M. Mosoiu, D. Rubio Bernabe, S. Scherg, A. Consorti, F.	2023	Realist synthesis	International	To describe how internet-based education can be used in undergraduate medical palliative care education, in terms of realist theorisation.	Undergraduate student studies	The study aimed to address the following questions: • What are the elements to be considered in the educational design of online teaching for PC? • Which are the possible educational theories, and which one suits best to which context and for which outcomes? Pros and cons? • Which methods work well for any given learning outcome? • Which is the best way to adapt methods to a given context, considering its features?	N/A - wide variety of countries studied	The use of cognitivism (such as clinical exercise solving, case-based learning, and virtual case simulation) as a theoretical framework for planning palliative care education is beneficial. Positive findings were noted for the use of cognitive constructivism, where active learning engages students in high-order thinking, practical knowledge application, and multiparty involvement. The study also highlighted the importance of contextualisation, as consistently considering PC throughout the rest of the curriculum is beneficial. Regarding duration, long forms are preferred in many positively received interventions; however, shorter modules offer more flexibility.
I Like Palliative Care Now': Improving Confidence in Palliative Care Using Simulation for Foundation Year 2 Trainees	Massey, L. Robinson, L. Huggin, A. Churm, D.	2023	Review of intervention	United Kingdom	To introduce and evaluate a novel palliative medicine simulation session as a tool for Foundation Year 2 (FY2) doctors to gain competency and confidence in the assessment and management of life-limiting illness	Foundation Year 2 (FY2) doctors	Three simulated scenarios involved management of opioid toxicity, breaking bad news and shared decision making with a role-play patient with a GI bleed. The session was evaluated using a pre and post-session questionnaire, collecting data with 5-point Likert scales and free-text comments.	Doctors who are in their second year of training post qualification, based in DASH simulation suite in Wansbeck General Hospital, Ashington, United Kingdom	The use of simulation as part of palliative care education programmes can improve confidence and communication in patient management situations. The study identified that debriefing following simulation was one of the most helpful and crucial aspects of this intervention.
Introducing a Palliative Care Simulation Day for Final Year Medical Students	Taylor, A. Doherty, C. Stone, C.	2022	Journal	United Kingdom	To assess the use of introducing a palliative care simulation week into the undergraduate curriculum, incorporating blended learning of communication skills, simulation, and clinical immersion.	Final year medical students	Four simulation scenarios on opioid toxicity, breathlessness, constipation, and agitation were used. Each scenario included a debrief and opportunity for repeat simulation, reinforcing skills and boosting confidence. The feedback from the sessions was then reviewed and assessed.	Centre for Medical Education, Queen’s University Belfast; Marie Curie Hospice, Belfast	Three key conclusions were drawn: the importance of expert advice and trial simulation scenarios, facilitator training—especially in debriefing—and opportunities to repeat simulations. The multidisciplinary approach used in this study also highlighted the significance of effective interprofessional competency and collaboration.
Understanding medical students' perception of end-of-life care	Ujoodha, T. Kulkarni, S. Fonseca, A.	2022	Conference abstract	United Kingdom	Two key aims were identified: • To create an objective questionnaire to analyse the understanding and experience of end-of-life care among medical students • To identify gaps in their learning and experience during their clinical placement	3rd and 5th year medical students	An objective, anonymous questionnaire was administered to a select group of medical students during their ICU placement. The questions looked at current/previous experience, perception, relevant university/ placement teaching of end-of-life care and management of psychological impact. The results were then collated to determine the viewpoints of the medical students regarding palliative care.	Critical Care Unit, Royal Preston Hospital, Fulwood, United Kingdom	Although 95% students agreed that end-of-life care is critical, there was a significant disparity in the experiences and practical application of teaching whilst on placement. The study recommended that medical students be involved in end-of-life treatment decisions by coordinating existing formal teaching or e-learning materials, which should be consolidated during their placement. The study also suggested providing psychological support in the form of debrief sessions with various stakeholders such as clinical supervisors, learner support and the medical school.
Care of the dying - Medical student confidence and preparedness: Mixed-methods simulation study	Wells, G. Llewellyn, C. Hiersche, A. Minton, O. Barclay, D. Wright, J.	2022	Mixed methods interventional study	United Kingdom	To explore whether the simulated experience of caring for a dying patient and their family can improve the confidence and preparedness of medical students to provide such care.	4th-year medical students	Simulation of caring for a dying patient was undertaken with serial measures of confidence using the Self-Efficacy in Palliative Care (SEPC) tool taken at pre, post and 6 months post-simulation time periods. Quantitative data were used to determine whether a novel simulated teaching intervention involving the care of a dying patient and their family could lead to a sustained increase in student confidence to provide such care. Qualitative data were used to explore how prepared students felt about caring for the dying.	Brighton and Sussex Medical School, Brighton, United Kingdom	The study deduced that simulated intervention leads to a sustained increase in the confidence of medical students to care for dying patients, particularly in the areas of communication skills, patient management and MDT working. Confidence in MDT working continued to increase during the 6-month post-simulation period. It also highlighted that students felt a lack of exposure to actively dying patients during clinical placements and that simulation would help address this by providing a consistent approach to teaching in this area.
Simulation to improve medical student confidence and preparedness to care for the dying: a feasibility study	Wells, G. Montgomery, J. Hiersche, A.	2022	Feasibility study	United Kingdom	To assess whether simulation can enhance the confidence and preparedness of medical students to provide comprehensive care to dying patients and their families, encompassing clinical assessment, symptom management, communication, and care after death.	4th-year medical students	Individual simulations involving a high-fidelity simulator (representing a dying patient) and an actor (playing a family member) were used. The pre-simulation/post-simulation thanatophobia questionnaires measured student attitudes towards providing care to dying patients. Thematic analysis of post-simulation focus group transcripts generated qualitative data regarding student preparedness, confidence and value of the simulations.	Brighton and Sussex Medical School, Brighton, United Kingdom	Results suggested a reduction in thanatophobia among the participants because of the simulations, with students feeling less uncomfortable, uneasy or helpless when thinking about looking after dying patients. It highlighted that the simulation environment felt safe and controlled, allowing students to make mistakes without fear. However, it also found that students wanted to repeat the simulation in the future to boost their confidence further. However, it did reveal that some students felt simulated environments were unrealistic for a typical doctor's experience and pressures.
GMC Outcomes for graduates	General Medical Council (GMC)	2018 (reviewed annually)	Literature	United Kingdom	To outline the standards and requirements for all stages of medical education and training in the United Kingdom	Designed for medical students, newly qualified doctors, medical schools and the GMC to ensure a standardised set of expectations for the standards required for newly qualified doctors.	The guide is: • For students to know what they need to learn during their time at medical school • Basis for medical schools to develop their curricula and programmes of learning • A blueprint or plan for assessments at medical schools • A framework that the GMC uses to regulate medical schools • A summary of what newly qualified doctors will know and be able to do for those designing postgraduate training	Medical students, newly qualified doctors and medical schools in the United Kingdom	It describes how newly qualified doctors must: • Recognise the complex medical needs, goals and priorities of patients, including a patient’s psychological and sociological factors • Be able to make decisions to reduce the burden of treatment where appropriate in patients with multiple conditions or who are approaching the end of life. It is crucial to understand the need to involve patients, their relatives, carers or other advocates in management decisions, making referrals and seeking advice from colleagues as appropriate. • Evaluate the clinical complexities, uncertainties, emotional challenges and uncertainty of diagnosis and treatment success involved in caring for end-of-life patients and demonstrate the relevant communication to be used with the patient, their relatives, carers or other advocates. • Be able to recognise when a patient is deteriorating and take appropriate action. • Recognise the challenges of safe prescribing for patients at the end of life
Association for Palliative Medicine Curriculum for undergraduate medical education (2025) Version 3	Barclay, S. Ellershaw, J.	2024	Literature	United Kingdom	To provide a framework for the learning outcomes expected of newly qualified doctors in providing care to palliative and end-of-life patients.	Undergraduate medical school education teams, as well as medical students and newly qualified doctors	Using the GMC 'Outcomes for Graduates' 2018, learning outcomes regarding junior doctors' knowledge, skills and attitudes towards palliative care are laid out.	Education systems and individuals with it, based in the United Kingdom.	The document focuses on the knowledge, skills and attitudes in 9 key areas that undergraduate medical education should include in relation to palliative care: • Basic principles • Physical care in relation to disease processes, symptom management, pain, other symptoms and care of the dying patient • Psychosocial care • Communication with patients, relatives and colleagues • Social and family relationships • Grief and bereavement • Personal and professional issues • Culture, language, religious and spiritual issues • Ethical and legal issues

### Protocol and registration

The protocol has been registered on the OSF register (DOI: 10.17605/OSF.IO/6J3CS). The scoping review strictly aligned with the JBI methodology [14] and is reported methodically using the PRISMA-ScR checklist (Appendix II).

## Results

### Study characteristics

Ten publications were included, comprising eight primary studies and two grey literature sources. Three papers were international studies [15–17], including one from the UK, and the remaining five were conducted in the UK [18–24]. The populations studied were predominantly medical students [18–22]; one study also included nursing students [15], and another included medical students, physicians, and nurses [16]. The two grey literature sources were aimed at medical students and those designing or running medical school curricula (GMC *Outcomes for Graduates* [23] and *Association for Palliative Medicine Curriculum for Undergraduate Medical Education 2025* [24]). The studies varied in their objectives but were all related to palliative care education; four specifically focused on the use of simulation [18, 22], one looked at medical students’ perspectives on end-of-life care [20], one assessed the use of online learning [17], one reviewed assessment models [16], one looked at various teaching methods [15] and the two grey literature sources provided guidelines for the medical school curriculum [23, 24].

### Thematic analysis

The analysis enabled the synthesis of the evidence to generate six thematic areas of focus, from which QIs can be developed:

- Undergraduate palliative care education to be made compulsory within the undergraduate medical curriculum.

The evidence highlights the importance of integrating palliative care education into the undergraduate curriculum. This concept is aptly demonstrated by Hokka et al., who reports that “education is seen as a facilitator to develop the integration of palliative care” [15] in preparing students for clinical practice, and supported by Ujoodha et al., who explained that “we (as clinicians) play an important role in preparing students to face and care for the terminally ill” [20]. Integrating palliative care education into the curriculum is not only essential to the general training of doctors but also part of a structural and organisational obligation to ensure that patients with life-limiting illness are cared for appropriately. Students are also aware of the challenges they will face in practice in supporting patients at the end of life. Wells et al. [21] note that Gajebasia et al., report that “junior doctors often desire greater representation of palliative medicine teaching within undergraduate curricula” [25], and Massey reports that only “27% of FY2s felt that their training so far had prepared them to deal with Palliative Care issues” [18]. Hence, the drive for improved and standardised palliative care education stems from educational and clinical needs, as well as from the concerns of junior doctors, who often feel a lack of competence and training in this field. Therefore, mandating that medical schools include palliative care in their curricula, preferably through a national set of objectives and minimum placement hours, would help prepare doctors to treat end-of-life patients upon qualification.

b) Mandatory assessment of palliative care in the undergraduate curriculum.

The integration of palliative care into medical education must be accompanied by rigorous assessment strategies to ensure that curricular content translates into clinical competence. Lopez-Garcia et al. report that structured assessments of knowledge and skills can effectively identify limitations in clinical practice [16]. Their analysis of various assessment tools highlights the crucial role of evaluation in tracking learner progress, encouraging reflective practice, and informing curriculum development. To maintain educational relevance, educators must critically appraise the format and validity of assessment instruments and align them with current evidence and evolving standards in palliative care education.

c) Introduction of palliative care-specific simulation sessions.

The importance of using simulation as a specific educational method was emphasised in five [15, 22] of the ten publications. Simulation prepares medical students for clinical practice by providing a psychologically safe environment in which learners can experiment, make mistakes, and receive feedback without risking patient safety. This controlled simulation setting encourages reflection in practice, enhances clinical reasoning, and builds confidence through repeated exposure to realistic scenarios. For example, Hokka et al., report how “simulations decreased students’ fears of causing addiction” when adjusting medication at the end of life [15]. Similarly, Massey et al. highlight that “95.6% of FY2s felt the session addressed the challenges they experienced managing PC issues” [18]. Wells et al., explored how simulation “led to a sustained increase in the confidence of medical students to care for dying patients” [21] and that “students coveted the exposure afforded to them by the simulations” [21]. The feedback from simulations revealed that “students commented on the desire to repeat the simulation at a future date in a bid to improve confidence further” [22], highlighting the desire of students to reflect and adapt their simulation skills through repetition.

d) Ensuring an interdisciplinary education with exposure to MDT working

Several publications support the view that medical students gaining exposure and experience within an MDT is crucial for developing and understanding a holistic approach to palliative care. For example, Hokka et al. classed having MDT experience as an “essential” [15] component, and Taylor et al. highlighted “the significance of effective interprofessional competency and collaboration” [19]. GMC *Outcomes for Graduates* stipulate that newly qualified doctors must be able to “demonstrate working collaboratively with other health and care professionals and organisations when working with patients, particularly those with multiple morbidities” [23] and in the APM curriculum frequently comments on the “role of doctors as part of the wider multidisciplinary team” [24] in the context of palliative care. These findings demonstrate that early clinical exposure in witnessing and working with various MDT members leads to more effective collaboration and an improved patient experience.

e) Implementation of clinical placements in palliative care settings

A clinical rotation in a palliative care setting, such as a hospice or a specialist hospital department, can help contextualise the application of core teaching content to clinical practice. For example, Hokka et al. reported that “elective courses increased students’ knowledge of palliative care” [15]. Ujoodha et al. emphasise the necessity of this, reporting that formal teaching must be “consolidated on the shopfloor … three quarters of the students have not (previously) witnessed any EOL discussions with patients or their family” [20]. Findings, such as those from Ujoodhe, highlight the significant gap between education and clinical exposure. Additionally, GMC *Outcomes for Graduates* [23] requires that medical schools ensure high-quality placements that build students’ clinical knowledge, skills, and experience. Given that several GMC outcomes relate to providing palliative care to complex patients [24], it can be argued that specialist clinical placements are imperative within the curriculum to ensure that junior doctors are sufficiently prepared. However, one key question not addressed in the literature is how long such rotations should be to ensure transfer of learning.

f) Use of IT and e-learning to support traditional and clinical teaching methods

E-learning, simulation, and IT-enhanced platforms support traditional and clinical teaching by providing a safe, controlled environment in which students can practice clinical decision-making, receive immediate feedback, and engage in iterative learning. These tools encourage experimentation, can reduce performance anxiety, and help bridge the gap between theoretical knowledge and clinical practice. For example, Hokka et al. explained that the “use of computer-based decision aid … and educational games … were effective” [15] as teaching methods. Moreover, Martucci et al. suggested that “the use of technology in simulation … is more effective” [17] in certain situations and has numerous advantages, including “consistency of content delivery, convenience, flexibility, addressing topics for which there is no local expertise, and reviewing content in a ‘just-in-time’ fashion” [17], and being especially “useful when faculty resources are scarce” [17]. These studies highlight that IT can be used alongside traditional teaching to address some limitations of face-to-face learning and to enhance educational programs.

### Proposed quality indicators

Building on the six thematic areas and structured using the Donabedian model [10] as a framework, the following measurable quality indicators are proposed (Table 3). These indicators are designed to be practical tools for curriculum development and quality assurance.

**Table 3:** Updated list of recommended QIs for scoping review of quality indicators in palliative medicine education in the UK

**Theme**	**Proposed Measurable Quality Indicator (QI)**	**Donabedian Category**
Compulsory Curriculum Integration	QI 1: 100% of UK medical schools have a defined, mandatory palliative care curriculum that maps to the GMC's *Outcomes for Graduates* and the APM Undergraduate Curriculum. Measurement: Audit of medical school curriculum documents against a checklist derived from national standards.	Structure
Mandatory Assessment	QI 2: 100% of medical students are assessed on palliative care competencies through a validated tool (e.g., OSCE station, knowledge test, case-based discussion) before graduation. Measurement: Review of assessment blueprints and pass/fail rates for palliative care components in summative exams.	Process
Simulation-Based Education	QI 3: ≥90% of final-year students report increased confidence in core palliative care skills (e.g., breaking bad news, symptom management) after participating in a simulated scenario. Measurement: Pre- and post-simulation evaluation using validated assessment tools.	Process/Outcome
Interdisciplinary Education	QI 4: 100% of medical schools provide a documented opportunity for students to learn with or from at least two other palliative care professional groups (e.g., specialist nurses, physiotherapists, chaplains) during their training. Measurement: Analysis of timetable and placement structures; student feedback on interprofessional learning experiences.	Process
Clinical Placements	QI 5: ≥80% of students complete a clinical placement that includes direct exposure to patients with specialist palliative care needs (e.g., in a hospice, hospital palliative care team, or community setting).Measurement: Analysis of student placement records and logs; surveys on quantity and quality of clinical exposure.	Process
E-Learning & IT Support	QI 6: A centrally maintained, evidence-based e-learning resource on core palliative care topics is available to 100% of students, with ≥75% completion rate for mandatory modules. Measurement: Analytics from virtual learning environment (VLE) tracking logins and module completion; integration with curriculum mapping	Process/Structure

## Discussion

Despite the increasing demand for palliative care and its recognition as a core component of holistic medical practice (WHO), its integration into undergraduate medical education in the UK remains inconsistent, fragmented, and insufficiently evaluated [26]. However, this scoping review identified and synthesised potential quality indicators (QIs) to guide the development and assessment of palliative care education in UK medical schools. By aligning findings with the Donabedian model of healthcare quality, the proposed QIs offer a structured framework for embedding palliative care into curricula in a manner that is measurable and responsive to learner needs.

### Key findings

Compulsory curriculum integration was consistently identified as a foundational element. The reviewed studies highlighted the variability in exposure to palliative care across UK medical schools and the resulting disparities in preparedness among junior doctors. Embedding palliative care as a core, mandatory component of the curriculum, aligned with the GMC’s Outcomes for Graduates [23] and the APM curriculum [24], would ensure a baseline of competence and confidence in managing end-of-life care.

Mandatory assessment was also emphasised as a mechanism to ensure accountability and reinforce learning. Validated tools and assessment strategies, such as OSCEs and knowledge tests, can be used to evaluate student competencies. Data suggest that the absence of assessment may lead to palliative care being perceived as peripheral rather than essential.

Simulation-based education emerged as a particularly effective pedagogical tool. Multiple studies demonstrated that simulation enhances student confidence, communication skills, and preparedness for emotionally complex scenarios. In parallel, multiprofessional and interdisciplinary education and exposure were highlighted as essential for preparing students to work collaboratively in clinical settings.

Clinical placements in palliative care settings were shown to bridge the gap between theoretical knowledge and practical application. However, the review also revealed a lack of consistency in placement availability and duration. E-learning and IT-enhanced education are identified as valuable adjuncts to traditional teaching. These tools offer flexibility, scalability, and consistency, particularly in resource-limited settings.

The six proposed QIs provide a practical roadmap for standardising palliative care education. Their development from the thematic analysis ensures that the Qis are grounded in the current evidence base. For curriculum developers and regulatory bodies such as the GMC, these QIs provide a preliminary set of standards to inform curriculum development, and identify standards against which curricula can be audited and improved.

### Quality Indicators

From this review, a defined set of QI’s have been suggested based on the Donabedian model, wherein; structure indicators refer to the institutional and curricular frameworks (e.g., presence of a defined palliative care curriculum), process indicators reflect the educational activities (e.g., simulation, assessment, placements), and outcome indicators relate to learner development and patient care (e.g., improved confidence, communication, and clinical competence). The use of this model supports a systems-level approach to curriculum design and evaluation, ensuring that educational interventions are not only implemented but also assessed for impact.

### Implications for practice

The findings of this review have direct implications for curriculum developers, medical educators, and regulatory bodies. The proposed QIs offer a practical, standardised yet adaptable roadmap for embedding palliative care in undergraduate education. Medical schools should consider auditing their curricula against these indicators and integrating them into quality assurance processes. National bodies such as the GMC and APM could support this by endorsing a minimum set of QIs and encouraging their adoption across institutions. However, implementation will require careful consideration of local contexts, faculty development, and resource allocation. Barriers such as limited placement availability, faculty expertise, and curricular space must be addressed through strategic planning and collaboration across institutions. Future research must focus on rigorously developing and piloting these specific QIs. This should include establishing their feasibility, reliability, and validity in real-world educational settings. The ongoing EU-funded CODE-YAA@PC-EDU project [27] is a promising example of the collaborative work needed in this area.

### Limitations of the study

Although UK-based studies were prioritised, three international papers were included as they either described the UK undergraduate palliative education system or referenced global online systems for implementing palliative education, which are applicable to UK palliative curriculum developers. This improves the research’s applicability and methodological strength, whilst remaining within the strict inclusion criteria for relevance to UK medical education. These studies addressed transferable themes, such as simulation, assessment tools, and e-learning, that are already present in UK curricula. Their inclusion enriched the thematic synthesis and supported the development of robust, evidence-based quality indicators applicable to UK settings.

The reliance on some unpublished or abstract-only sources [18–20] is another limitation, as it restricted the depth of analysis for those contributions. The authors of these three sources were contacted to inquire whether a full-text version was available, and following this, received confirmation that these abstracts were never developed further into published papers.

Although QIs are a relatively new concept, they can be used by medical programme developers to effectively mould the curriculum and establish measurable outcome goals. Given that QIs are a set of preliminary indicators derived from evidence synthesis, their implementation requires further testing and development through consensus methodology and validation to make them applicable for use. A Delphi study, for example, using multiple iterations of the QIs to form a collated set of domains for future indicator development, would be required to apply these principles practically within the curriculum.

## Conclusion

This scoping review has synthesised the current evidence to propose a set of six measurable quality indicators for undergraduate palliative care education in the UK. These QIs, structured around the themes of compulsory integration, assessment, simulation, interdisciplinary learning, clinical placements, and e-learning, provide a proposed framework for enhancing educational quality and standardisation. The findings underscore a clear consensus on the need to better prepare junior doctors for end-of-life care. Adopting and evaluating these QIs represents a critical next step in ensuring that all newly qualified doctors possess the necessary competence and confidence in this fundamental aspect of medical practice.

## Data Availability

All papers in the review are accessible through the cited databases.

## Appendix

### Appendix I: search strategy for CENTRAL. Original search conducted on Dec/2024

**Table:**

Hashtag	Query	Results
#1	MeSH descriptor: [Palliative Care] explode all trees	2661
#2	Terminal care	2846
#3	Palliative care	8477
#4	End of life	45,426
#5	Hospice care	1402
#6	MeSH descriptor: [Hospice Care] explode all trees	187
#7	MeSH descriptor: [Terminal Care] explode all trees	782
#8	Medical education	36,170
#9	Teaching	24,860
#10	Curriculum	7689
#11	MeSH descriptor: [Education, Medical] explode all trees	4764
#12	MeSH descriptor: [Teaching] explode all trees	6085
#13	MeSH descriptor: [Curriculum] explode all trees	2777
#14	MeSH descriptor: [Training Support] explode all trees	68
#15	MeSH descriptor: [Fellowships and Scholarships] explode all trees	56
#16	MeSH descriptor: [Faculty, Medical] explode all trees	136
#17	MeSH descriptor: [Medical Staff] explode all trees	460
#18	Medical staff	12,542
#19	Physician	47,808
#20	Doctor in training	2314
#21	Foundation year doctor	499
#22	Foundation year medic	36
#23	New doctor	2937
#24	Newly qualified doctor	50
#25	Foundation year	19,617
#26	Medical student	11,095
#27	MeSH descriptor: [Physicians] explode all trees	3993
#28	MeSH descriptor: [Students, Medical] explode all trees	1878
#29	FY1	7
#30	FY2	7
#31	Senior house officer	31
#32	Registrar	427
#33	Junior doctor	141
#34	(#1 OR #2 OR #3 OR #4 OR #5 OR #6 OR #7)	53,806
#35	(#8 OR #9 OR #10 OR #11 OR #12 OR #13 OR #14 OR #15)	62,745
#36	(#16 OR #17 OR #18 OR #19 OR #20 OR #21 OR #22 OR #23 OR #24 OR #25 OR #26 OR #27 OR #28 OR #29 OR #30 OR #31 OR #32 OR #33)	91,173
#37	(#34 AND #35 AND #36)	440

Supplemental search conducted 25/03/2026, spanning 14/12/2024 to 25/03/2026

**Table:**

Databases	Hits	Duplicates	Screened	Included in Review
Medline, Embase & Emcare	167	53	114	0
Cochrane	87	0	87	0

### Appendix II: PRISMA-ScR checklist

**Table:**

SECTION	ITEM	PRISMA-ScR CHECKLIST ITEM	REPORTED ON PAGE #
TITLE			
Title	1	Identify the report as a scoping review.	2
**ABSTRACT**			
Structured summary	2	Provide a structured summary that includes (as applicable): background, objectives, eligibility criteria, sources of evidence, charting methods, results, and conclusions that relate to the review questions and objectives.	2
**INTRODUCTION**			
Rationale	3	Describe the rationale for the review in the context of what is already known. Explain why the review questions/objectives lend themselves to a scoping review approach.	3
Objectives	4	Provide an explicit statement of the questions and objectives being addressed with reference to their key elements (e.g., population or participants, concepts, and context) or other relevant key elements used to conceptualize the review questions and/or objectives.	3–4
**METHODS**			
Protocol and registration	5	Indicate whether a review protocol exists; state if and where it can be accessed (e.g., a Web address); and if available, provide registration information, including the registration number.	7
Eligibility criteria	6	Specify characteristics of the sources of evidence used as eligibility criteria (e.g., years considered, language, and publication status), and provide a rationale.	3–5
Information sources*	7	Describe all information sources in the search (e.g., databases with dates of coverage and contact with authors to identify additional sources), as well as the date the most recent search was executed.	4–5
Search	8	Present the full electronic search strategy for at least 1 database, including any limits used, such that it could be repeated.	26
Selection of sources of evidence†	9	State the process for selecting sources of evidence (i.e., screening and eligibility) included in the scoping review.	5–7
Data charting process‡	10	Describe the methods of charting data from the included sources of evidence (e.g., calibrated forms or forms that have been tested by the team before their use, and whether data charting was done independently or in duplicate) and any processes for obtaining and confirming data from investigators.	6–7
Data items	11	List and define all variables for which data were sought and any assumptions and simplifications made.	5–7
Critical appraisal of individual sources of evidence§	12	If done, provide a rationale for conducting a critical appraisal of included sources of evidence; describe the methods used and how this information was used in any data synthesis (if appropriate).	Not applicable
Synthesis of results	13	Describe the methods of handling and summarizing the data that were charted.	6–7
**RESULTS**			
Selection of sources of evidence	14	Give numbers of sources of evidence screened, assessed for eligibility, and included in the review, with reasons for exclusions at each stage, ideally using a flow diagram.	5–7
Characteristics of sources of evidence	15	For each source of evidence, present characteristics for which data were charted and provide the citations.	7
Critical appraisal within sources of evidence	16	If done, present data on critical appraisal of included sources of evidence (see item 12).	Not applicable
Results of individual sources of evidence	17	For each included source of evidence, present the relevant data that were charted that relate to the review questions and objectives.	8–12
Synthesis of results	18	Summarize and/or present the charting results as they relate to the review questions and objectives.	13–19
**DISCUSSION**			
Summary of evidence	19	Summarize the main results (including an overview of concepts, themes, and types of evidence available), link to the review questions and objectives, and consider the relevance to key groups.	16–19
Limitations	20	Discuss the limitations of the scoping review process.	19
Conclusions	21	Provide a general interpretation of the results with respect to the review questions and objectives, as well as potential implications and/or next steps.	20
**FUNDING**			
Funding	22	Describe sources of funding for the included sources of evidence, as well as sources of funding for the scoping review. Describe the role of the funders of the scoping review.	21
